# Case Report: Prolonged clinical benefit with sequential trastuzumab-containing treatments in a patient with advanced extramammary Paget disease of the groin

**DOI:** 10.3389/fonc.2022.925551

**Published:** 2022-08-18

**Authors:** Emma Zattarin, Federico Nichetti, Francesca Ligorio, Laura Mazzeo, Riccardo Lobefaro, Giovanni Fucà, Giorgia Peverelli, Andrea Vingiani, Giulia V. Bianchi, Giuseppe Capri, Filippo de Braud, Claudio Vernieri

**Affiliations:** ^1^ Department of Medical Oncology, Fondazione IRCCS Istituto Nazionale dei Tumori, Milan, Italy; ^2^ Istituto Fondazione di Oncologia Molecolare Ente del Terzo Settore, The AIRC Institute of Molecular Oncology, Milan, Italy; ^3^ Department of Pathology and Laboratory Medicine, Fondazione IRCCS Istituto Nazionale dei Tumori, Milan, Italy; ^4^ Department of Oncology and Hemato-Oncology, University of Milan, Milan, Italy

**Keywords:** extramammary Paget disease (EMPD), HER2 overexpression, trastuzumab, prolonged benefit, antiandrogen therapy, rare cancer

## Abstract

Extramammary Paget disease (EMPD) is a rare form of cutaneous, intraepithelial adenocarcinoma, which typically presents itself as an erythematous plaque originating from apocrine-gland rich regions, such as the vulva, the perianal region, the scrotum, the penis, or the axilla. EMPD patients typically have a good prognosis, with expected 5-year survival of 60%–92%, but it is estimated that about one-third of EMPD patients will develop lymph node or distant metastases. Treatment approaches for EMPD include locoregional therapies such as broad surgical resection, radiotherapy, or topical imiquimod, when the disease is localized, and chemotherapy and biological agents for advanced EMPD. We report the case of a 58-year-old man diagnosed with locally advanced, symptomatic HER2-overexpressing, AR-positive EMPD, who achieved long-term tumor control with a sequence of several trastuzumab-based treatments (more than 30 months with second-line carboplatin plus paclitaxel plus trastuzumab followed by trastuzumab maintenance; 9 months for third-line vinorelbine plus trastuzumab). Even if it is reported that AR expression occurs concomitantly with HER2 overexpression in more than half of the cases of EMPD, to the best of our knowledge, this is the first case report describing androgen receptor blockade therapy in combination with an anti-HER2 agent. Our patient did not benefit from androgen receptor blockade in combination with trastuzumab, thus suggesting that AR expression may simply reflect an intrinsic characteristic of the EMPD cell of origin, rather than tumor dependence upon AR signaling. Given the reported sensibility to anti-HER2 therapy, also new antibody drug conjugates targeting HER2 are worth exploring in the management of advanced EMPD.

## Introduction

Extramammary Paget disease (EMPD) is a rare form of cutaneous, slowly growing intraepithelial adenocarcinoma originating from apocrine-gland rich regions, such as the vulva, the perianal region, the scrotum, the penis, or the axilla (1). Similarly to the more common mammary Paget disease (MPD), EMPD typically presents itself as an erythematous plaque that can evolve as erosive, crusty, or eczematous. Clinical features of EMPD resemble inflammatory, non-malignant conditions, thus frequently resulting in misdiagnoses or delayed recognition of the cancerous nature of this disease. EMPD more frequently affects female individuals, with a male-to-female ratio of 1:2.8 in Europe (2), and median age at diagnosis is 65 both in men and in women (3, 4). The overall incidence ranges from 0.1 to 2.4 patients per 1,000,000 person-years (5).

EMPD patients typically have a good prognosis, with expected 5-year survival of 60%–92% (5, 6). However, when EMPD invades the derma, it can spread to loco-regional lymph nodes and/or distant organs (7). It is estimated that about one-third of EMPD patients will develop lymph node involvement or distant metastases (8). In these cases, the expected 5-year survival is <10% (6). As in the case of Paget disease of the breast, human epidermal growth receptor 2 (HER2) overexpression is reported in up to 50% of EMPD patients, and it is associated with a more aggressive clinical behavior, including more frequent dermal invasion and lymph node spread (9). Androgen receptor (AR) is also frequently expressed in EMPD (50%–80% of cases), and similarly to HER2 overexpression, its expression is associated with the presence of invasive disease and more frequent metastatic spread (10–13).

Treatment approaches for localized EMPD include broad surgical resection, radiotherapy, topical imiquimod, and photodynamic therapy; for patients with advanced (locally advanced or metastatic) EMPD, several chemotherapeutic agents, including 5-fluorouracil, taxanes, platinum salts, and vinca alkaloids, have shown moderate antitumor activity in published case series (14–17). More recently, anti-HER2 agents, alone or combined with chemotherapy, have shown promising antitumor activity in patients with HER2-overexpressing EMPD, as summarized in **Table 1** (1, 9, 18–28). Androgen blockade therapy also showed some activity in few patients with EMPD expressing AR (27, 28).

**Table 1:** Available case series of locally advanced or metastatic HER2-positive EMPD treated with anti-HER2 therapies alone or in combination.

First author	Year of publication	Number of patients	Primary site of EMPD	Inguinal lymph node involvement	Sites of distant metastases	Anti-HER2 therapy	Chemotherapy in combination with anti-HER2 drug	PFS with the anti-HER2-based therapy treatment (months)
**Karam (** 1 **)**	2008	1	vulva	/	/	trastuzumab	/	14
**Hanawa (** 18 **)**	2011	1	vulva	yes	axillary and abdominal lymph nodes	trastuzumab	paclitaxel	12
**Wakabayashi (** 19 **)**	2012	1	vulva	yes	abdominal lymph nodes, lung, liver	trastuzumab	/	20
**Gunvén (** 20 **)**	2012	1	vulva	yes	abdominal lymph nodes, bone	trastuzumab	vinorelbine	36
**Barth (** 21 **)**	2015	1	scrotum	no	cervical lymph nodes, abdominal lymph nodes, bone	trastuzumab	/	>12
**Ichiyama (** 22 **)**	2017	1	vulva	/	/	trastuzumab	paclitaxel	>32
**Hsieh (** 9 **)**	2019	1	vulva	yes	/	trastuzumab	carboplatin + paclitaxel	7
**Hsieh (** 9 )*	2019	1	vulva	yes	/	T-DM1	/	6
**Lu (** 23 **)**	2019	2	scrotum	yes	abdominal lymph nodes; bone and liver	trastuzumab	/; paclitaxel	17; 5
**Nordmann (** 24 **)**	2019	1	scrotum	yes	thoracic and abdominal lymph nodes, lung	lapatinib	/	primary resistance
**Nordmann (** 24 **)**	2019	1	scrotum	yes	thoracic and abdominal lymph nodes, lung	trastuzumab	carboplatin	7.5
**Bartoletti (** 25 **)**	2020	4	vulva	yes	abdominal lymph nodes, lung pelvis; abdominal lymph nodes; anus	trastuzumab	paclitaxel	36; 10; 8; 16
**Bartoletti (** 25 )*	2020	1	vulva	yes	abdominal lymph nodes, lung	T-DM1	/	>4
**Kimura (** 26 **)**	2020	1	perianal	yes	abdominal lymph nodes	trastuzumab	/	>6

*After progression to first line anti-HER2 therapy. “;” separate the information from different patients.

Here, we report the case of a 58-year-old man diagnosed with locally advanced, symptomatic HER2-overexpressing, AR-positive EMPD, who experienced prolonged disease control when treated with subsequent lines of trastuzumab plus different cytotoxic agents.

## Case description

**Figure 1:**
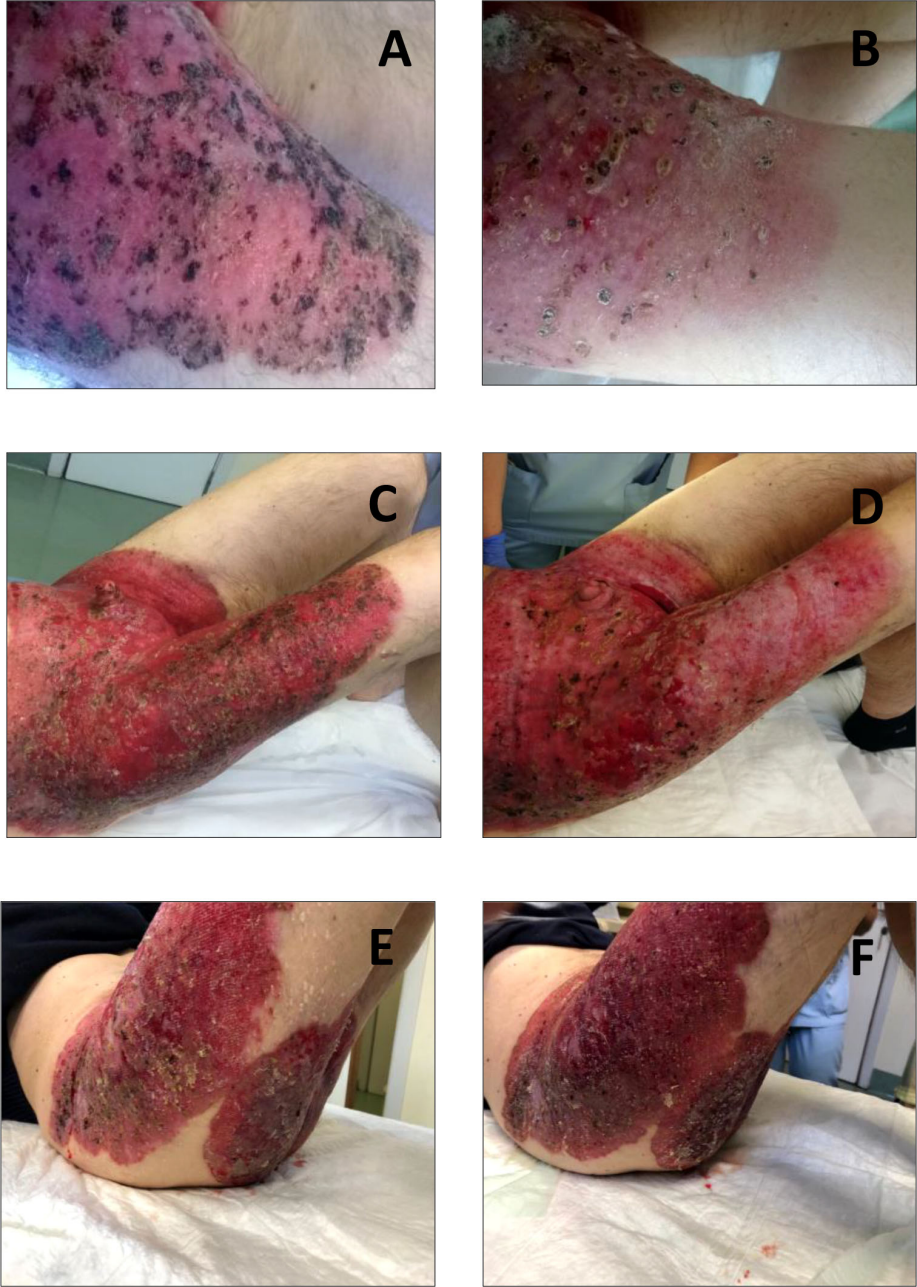
Skin scaly and erythematous plaques at different phases of the disease history of our patient. **(A)** Erythematous lesions with some ulcerated and bleeding areas at diagnosis. **(B)** Lesions appearing less erythematous and with initial signs of re-epithelization after 4 months of treatment with carboplatin, paclitaxel plus trastuzumab. **(C)** Clinical disease progression during trastuzumab maintenance therapy. **(D)** Reduction in bleeding and partial epithelialization of skin lesions after starting vinorelbine plus trastuzumab. **(E)** Clinical disease progression during vinorelbine plus trastuzumab. **(F)** Rapid disease progression during anti-androgen therapy plus trastuzumab.

**Figure 2:**
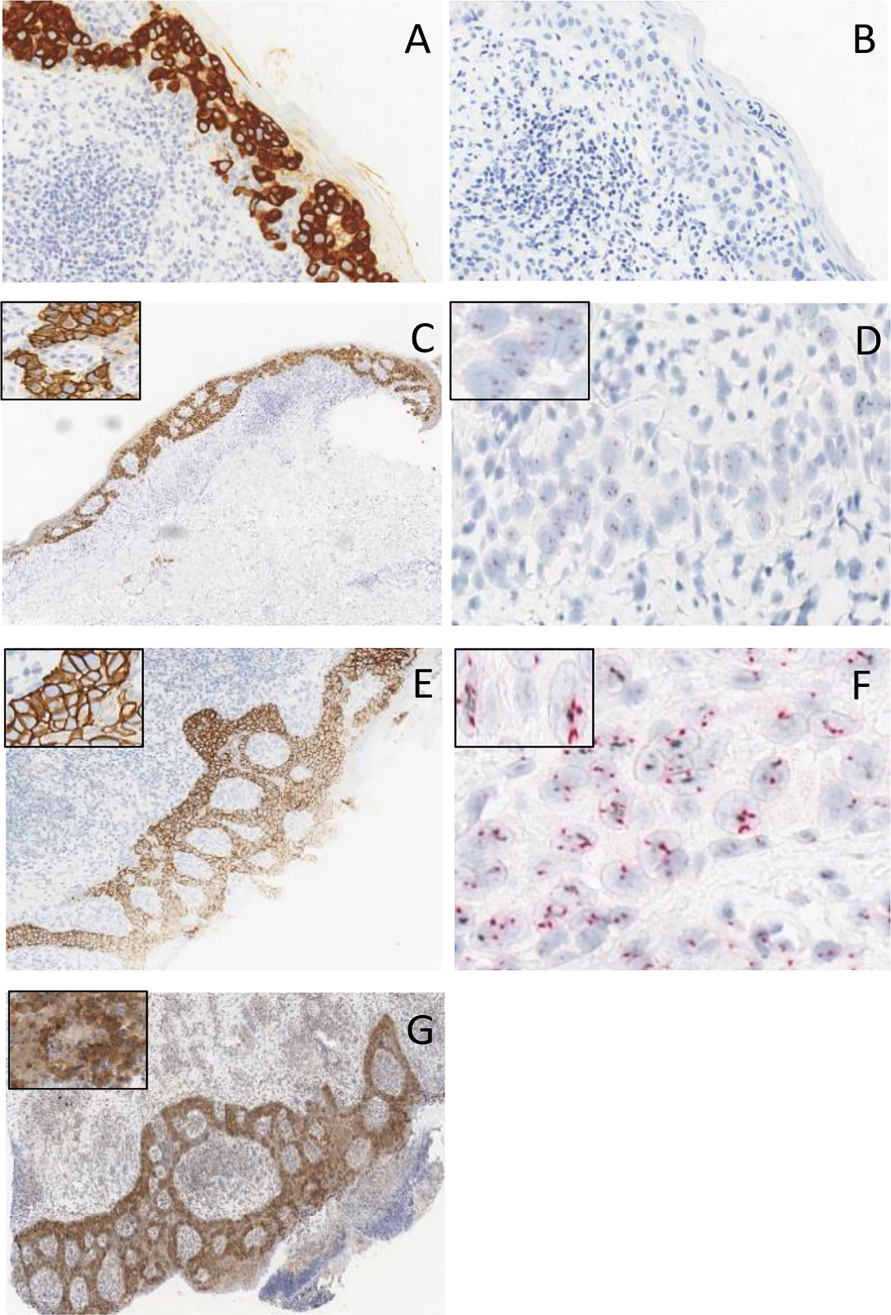
**(A)** Immunoreactivity for cytokeratin 7. **(B)** Negativity for cytokeratin 20. **(C, E)** HER2 strong membranous staining (3+) at immunohistochemical stain magnification, × 10, in two different tumor samples of the patient. **(D, F)** microscopic image in CISH for *HER2* staining in two different tumor samples of the patient. **(G)** Androgen receptor staining at immunohistochemical stain magnification, × 10.

**Figure 3:**
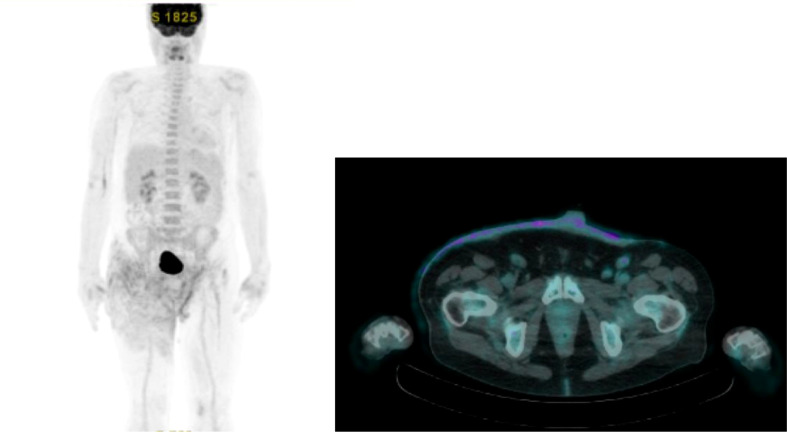
FDG PET/CT scan at baseline of patient’s clinical history.

**Figure 4:**
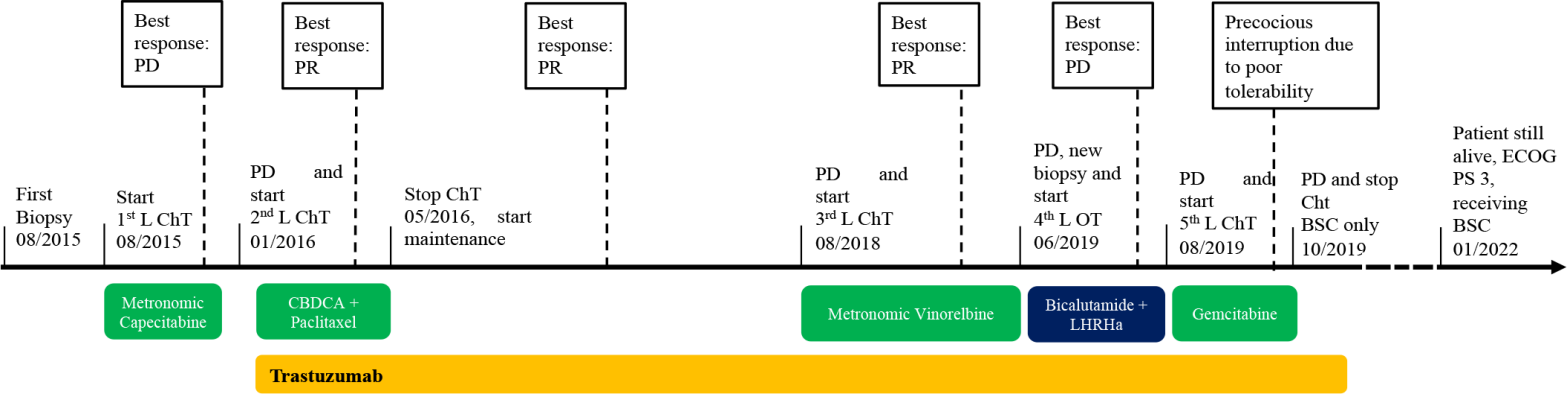
Timeline of systemic treatments administrated during patient’s disease history. BSC, best supportive care; CBDCA, carboplatin; ChT, chemotherapy; ECOG PS, Eastern Cooperative Group Perfomance Status; LHRHa, LHRH analogue; OT, hormonal therapy; PD, progression disease; PR, partial disease response.

In August 2015, a 58-year-old man with no significant comorbidities and a 3-year history of a non-healing groin rash, initially misdiagnosed as psoriasis that did not respond to topical antibiotics, antifungal agents, and steroids, was referred to our Institution. At the presentation, lesions were well-demarcated and widely extended over the skin from the umbilical line to both thighs, involving the groins, penis, scrotum, and perianal region. Some of these lesions were erythematous, with some ulcerated and bleeding areas, and others appeared as scaly and eczematous plaques, as shown in **Figure 1A**. The patient reported severe pain, itching, and dysuria (due to penis involvement). Skin punch biopsy revealed a proliferation of large round neoplastic elements spreading throughout the epidermis with dermal infiltration, coherent with EMPD. Immunoreactivity for cytokeratin 7 (**Figure 2A**) and EMA and negativity for cytokeratin 20 (**Figure 2B**) and Melan-A ruled out secondary pagetoid spread from urothelial or colorectal cancer and malignant melanoma (17). HER2 was highly expressed by immunohistochemistry (IHC) [score of 3+ according to CAP guidelines (29)] (**Figure 2C**), and *in situ* hybridization (ISH) analysis revealed *ERBB2* gene amplification (**Figure 2D**). Laboratory analyses revealed moderate anemia. A PET/CT scan showed avid fluorine-18-fluorodeoxy-D-glucose (FDG) uptake [maximum standardized uptake value (SUV max), 5] in correspondence with the cutaneous thickening of the suprapubic region, the perineum, and the right thigh, with bilateral inguinal enlarged lymph nodes (SUV max, 3.3), without metastases in visceral organs (**Figure 3**). Loco-regional approaches were excluded due to the extension of the disease, and the patient was started on systemic therapy. Different patient’s treatment lines are summarized in **Figure 4**. Local disease progression occurred after 4 months of first-line metronomic Capecitabine 1,500 mg/day in December 2015. The patient’s quality of life was highly compromised. He experienced a depressive mood, related to the disabling condition caused by the disease, with important limitations in terms of autonomy, mobilization, and need for hospital access for medication of skin lesions. The patient needed to take antidepressants but refused the psychological support that was offered to him.

Based on HER2 overexpression in tumor cells, the patient was candidate to receive anti-HER2-based therapy. Therefore, in January 2016, second-line treatment with carboplatin [area under the curve (AUC) of 5 every 3 weeks] plus paclitaxel (150 mg/mq every 3 weeks) in combination with trastuzumab (provided with an off-label procedure) was initiated. Trastuzumab was administered at a loading dose of 8 mg/kg i.v., followed by 6 mg/kg i.v. maintenance dose every 3 weeks, as for the treatment of HER2-positive breast cancer (30). Echocardiograms were performed prior to treatment start and every 3 months to assess patient’s cardiac function. After 4 months, the patient reported a good clinical response, with skin lesions appearing less erythematous and with initial signs of re-epithelization (**Figure 1B**). This was paralleled by remarkable symptomatic improvement, including reduction in itching, pain, bleeding, crusting, and overall disease extension. Chemotherapy was stopped after six cycles, while triweekly trastuzumab was continued as maintenance treatment for 32 months, with prolonged clinical benefit. During this period, patient symptoms and quality of life improved, and the need for skin lesion medication was reduced. In August 2018, when the patient was receiving trastuzumab maintenance, clinical disease progression occurred (**Figure 1C**), with worsening of pain and skin lesion bleeding causing anemia, which required blood transfusions. Then, third-line metronomic vinorelbine (oral vinorelbine at the dosage of 50 mg, as given on Monday, Wednesday, and Friday every week) was started, together with i.v. triweekly trastuzumab at 6 mg/kg dosage. The patient experienced clinical benefit from vinorelbine plus trastuzumab combination, with reduction in pain and bleeding and partial re-epithelialization of skin lesions, as shown in **Figure 1D** (as compared to **Figure 1C**). Unfortunately, metronomic vinorelbine treatment was poorly tolerated, with the occurrence of febrile neutropenia requiring prolonged hospitalization after 2 months of treatment. In January 2019, after hospital discharge, oral metronomic vinorelbine was resumed at a reduced dose (30 mg on Monday, Wednesday, and Friday every week) in combination with trastuzumab; treatment was continued until May 2019, when clinical disease progression occurred (**Figure 1E**).

Aiming to re-characterize tumor biology and to find potential therapeutic targets, we performed a new tumor biopsy to combine HER2 IHC/ISH evaluation and AR expression with the analysis of the hotspot regions of 50 cancer-related genes (Cancer Hotspot Panel v2; Thermo Fisher Scientific, Waltham, MA), as assessed by means of targeted next-generation sequencing (NGS) through the Ion Torrent Personal Genome platform “Hot-spot Cancer Panel” (Thermo Fisher Scientific). While confirming the overexpression/amplification of *HER2* (IHC/FISH) (**Figures 2E, F**), these evaluations also revealed the expression of AR in 90% of tumor cells (**Figure 2G**). NGS did not reveal mutations in targetable oncogenes. PCR amplification and direct sequencing of microsatellite loci revealed microsatellite stability.

Based on both AR expression and HER2 overexpression, in June 2019, fourth-line treatment with bicalutamide 50 mg p.o. daily, plus leuprorelin 11.25 mg i.m. every 3 months (according to the schedule commonly used in prostate cancer) was started, together with i.v. trastuzumab maintenance; however, the disease rapidly progressed (**Figure 1F**). In August 2019, endocrine therapy was interrupted and fifth-line treatment with gemcitabine (800 mg day 1, day 8 every 3 weeks) plus i.v. trastuzumab was initiated. The treatment was poorly tolerated, since the patient experienced febrile neutropenia and severe fatigue, which led to permanent treatment interruption after only two cycles. The patient was deemed unfit to tolerate further anticancer treatments, but he continued to receive palliative care.

As for March 2022, the patient is still alive, with a performance status of 3 by ECOG score, symptomatic for severe asthenia, and mobilization difficulties due to loco-regional cutaneous and nodal disease progression, which impair his walking capability and result in severe anemia and frequent blood transfusions. He is receiving daily best supportive care at home.

## Statement of patient consent

The patient provided written informed consent for his case to be presented.

## Discussion

Inoperable EMPD is typically associated with dismal prognosis, and systemic medical treatment is the only therapeutic choice. In the clinical case that we reported here, a patient with inoperable, loco-regionally advanced EMPD bearing HER2 overexpression (as a consequence of *HER2* gene amplification) achieved long-term tumor control with a sequence of several trastuzumab-based treatments (more than 30 months with second-line carboplatin plus paclitaxel plus trastuzumab followed by trastuzumab maintenance; 9 months for third-line vinorelbine plus trastuzumab).

Owing to its rarity, there is still limited available clinical evidence on the most effective treatment strategies for advanced EMPD, which indeed remains orphan of standard therapies. In the case of limited disease extension, non-surgical treatments, such as radiotherapy, photodynamic therapy, topical imiquimod, or carbon dioxide laser therapy, used alone or in combination, can be considered as valid and effective treatment options (17).

In case reports and case series published to date, chemotherapy has shown moderate antitumor activity (1, 24), and advanced EMPD remains associated with a dismal prognosis (31). With regard to biological therapies, several reports have clearly documented the efficacy of anti-HER2 therapies in HER2-positive EMPD, such as trastuzumab, alone and in combination with paclitaxel, or T-DM1 (**Table 1**). however, only in a few studies, trastuzumab-based therapies resulted in long-term tumor control, as described in our case. A Japanese phase II single-arm clinical trial enrolled 13 patients with advanced HER2-positive EMPD to receive docetaxel (75 mg/mq every 3 weeks) plus i.v. trastuzumab (UMIN000021311), but results have not been published yet. New antibody drug conjugates (ADCs) targeting HER2, like Trastuzumab-deruxtecan (T-DXd), recently demonstrated meaningful efficacy in HER2-positive advanced breast and gastric cancers and also showed preliminary activity in HER2-positive metastatic colorectal and non-small cell lung cancers (32–34). However, to our knowledge, T-DXd activity in EMPD remains unknown.

The only clinical trial currently enrolling patients with metastatic EMPD is a phase II, single-arm study offering the combination of nivolumab and ipilimumab to advanced rare tumors (NCT02834013). PD-L1 expression is typically low in most EMPDs (35). Nonetheless, a case report described a partial response to ipilimumab 1 mg/kg plus nivolumab 3 mg/kg, which lasted 7 months, in a patient with PD-L1-negative, MSI-stable, low tumor mutational burden metastatic EMPD (36).

The study of Liegl et al. reported that AR expression frequently occurs concomitantly with HER2 overexpression, both in MPD and EMPD (88% of MPD cases and 52% of EMPD cases) (37). Two case reports described a meaningfully decrease of multiple bone and lymph nodes metastases in two patients with AR-overexpressing, advanced EMPD treated with bicalutamide or chlormadinone acetate (anti-androgen drugs), respectively, combined with leuprorelin acetate (luteinizing hormone-releasing agonist) (27, 28). In our patient, combining androgen receptor blockade with trastuzumab did not provide benefit, thus suggesting that AR expression in EMPD cells does not necessarily imply tumor dependence upon AR signaling, nor it predicts response to anti-AR treatments, but it may simply reflect an intrinsic characteristic of the EMPD cell of origin, i.e., glandular cells with apocrine differentiation (38). Due to the high frequency of AR expression in EMPDs (11), future studies should focus on uncovering the determinants of EMPD dependence on the AR pathway to identify patients who could benefit from androgen blockade.

Dissimilarly to other EMPD case reports described in the literature, our patient never developed distant metastases. He presented with cutaneous involvement and bilateral inguinal lymphadenopathies and underwent several subsequent locoregional progression events responsible for a slow but progressive deterioration of clinical conditions. The absence of metastatic spread could be the result of intrinsic molecular characteristics of the tumor and/or of the antitumor and immunomodulatory effects of trastuzumab, which might explain the long-term disease control.

To the best of our knowledge, this is the first case reporting a prolonged clinical benefit and long-term survival after treatment with several trastuzumab-based regimens (i.e., four different treatment combinations, for a total of 45 months of trastuzumab-based therapies), including a trastuzumab–anti-androgen therapy combination, which has never been used in previous published EMPD case reports or case series. In patients with HER2-positive EMPD, the use of ADCs targeting HER2 should be further explored. In the context of a very rare disease, we suggest that sharing cases of patients achieving prolonged tumor control and long-term survival holds great value and might help clinicians in making decisions when managing these neoplasms.

## Data availability statement

The original contributions presented in the study are included in the article/supplementary material. Further inquiries can be directed to the corresponding author.

## Author contributions

EZ: conceptualization, manuscript preparing, editing. FN: conceptualization, manuscript preparing, editing. FL: manuscript preparing. LM: draft preparing. RL: manuscript preparing. GF: editing. GP: editing. AV: data collection. GB: conceptualization. GC: editing. FB: conceptualization. CV: conceptualization, manuscript preparing, editing, supervising. All authors contributed to the article and approved the submitted version.

## Acknowledgments

We would like to thank AIRC, the Associazione Italiana per la Ricerca sul Cancro, for funding our research (MFAG 22977: PI Claudio Vernieri).

## Conflict of interest

FB reports an advisory role at Roche, EMD Serono, NMS Nerviano Medical Science, Sanofi, MSD, Novartis, Incyte, BMS, Menarini; speaker role for BMS, Healthcare Research and Pharmacoepidemiology, Merck Group, ACCMED, Nadirex, MSD, Pfizer, Servier, Sanofi, Roche, AMGEN, Incyte, Dephaforum; Principal Investigator for Novartis, F.Hoffmann-LaRoche Ltd, BMS, Ignyta Operating INC, Merck Sharp and Dohme Spa, Kymab, Pfizer, Tesaro, MSD, MedImmune LCC, Exelixis Inc., LOXO Oncology Incorporated, DAICHI SANKIO Dev. Limited, Basilea Pharmaceutica International AG, Janssen-Cilag International NV, Merck KGAA. CV reports an advisory role for Novartis; travel grants: Lilly, Novartis, Istituto Gentili, Roche, Pfizer; research grants: Roche.

The remaining authors declare that the research was conducted in the absence of any commercial or financial relationships that could be construed as a potential conflict of interest.

## Publisher’s note

All claims expressed in this article are solely those of the authors and do not necessarily represent those of their affiliated organizations, or those of the publisher, the editors and the reviewers. Any product that may be evaluated in this article, or claim that may be made by its manufacturer, is not guaranteed or endorsed by the publisher.
